# Compound climate-pollution extremes in Santiago de Chile

**DOI:** 10.1038/s41598-023-33890-w

**Published:** 2023-04-25

**Authors:** Sarah Feron, Raúl R. Cordero, Alessandro Damiani, Pedro Oyola, Tabish Ansari, Juan C. Pedemonte, Chenghao Wang, Zutao Ouyang, Valentina Gallo

**Affiliations:** 1 Universidad de Santiago de Chile, Av. Bernardo O’Higgins 3363, Santiago, Chile; 2 University of Groningen, Wirdumerdijk 34, 8911 CE Leeuwarden, The Netherlands; 3 Center for Environmental Remote Sensing, Chiba University, 1-33 Yayoicho, Inage Ward, Chiba, 263-8522 Japan; 4 Centro Mario Molina, Antonio Bellet 292, Santiago, Chile; 5 Research Institute for Sustainability – Helmholtz Centre Potsdam (RIFS), Berliner Str. 130, 14467 Potsdam, Germany; 6 School of Medicine, Pontificia Universidad Católica de Chile, Santiago, Chile; 7 School of Meteorology & Department of Geography and Environmental Sustainability, University of Oklahoma, 120 David L. Boren Blvd. Suite 5220, Norman, OK 73072 USA; 8 Department of Geography and Environmental Sustainability, University of Oklahoma, Norman, OK 73019 USA; 9 Department of Earth System Science, Stanford University, Stanford, CA 94305-2210 USA

**Keywords:** Climate-change impacts, Atmospheric science

## Abstract

Cities in the global south face dire climate impacts. It is in socioeconomically marginalized urban communities of the global south that the effects of climate change are felt most deeply. Santiago de Chile, a major mid-latitude Andean city of 7.7 million inhabitants, is already undergoing the so-called “climate penalty” as rising temperatures worsen the effects of endemic ground-level ozone pollution. As many cities in the global south, Santiago is highly segregated along socioeconomic lines, which offers an opportunity for studying the effects of concurrent heatwaves and ozone episodes on distinct zones of affluence and deprivation. Here, we combine existing datasets of social indicators and climate-sensitive health risks with weather and air quality observations to study the response to compound heat-ozone extremes of different socioeconomic strata. Attributable to spatial variations in the ground-level ozone burden (heavier for wealthy communities), we found that the mortality response to extreme heat (and the associated further ozone pollution) is stronger in affluent dwellers, regardless of comorbidities and lack of access to health care affecting disadvantaged population. These unexpected findings underline the need of a site-specific hazard assessment and a community-based risk management.

## Introduction

Extreme heat has become more frequent and more intense across most land regions due to human-induced climate change^1–3^. Heatwaves, understood as periods of consecutive days with warmer than usual conditions, are already impacting health in a myriad of ways, including by leading to death and illness^4,5^. Excessive heat can increase the likelihood of heat stroke or dehydration, especially in vulnerable populations without access to adequate cooling mechanisms and in residents with pre-existing medical conditions that reduce heat endurance^5^.

Climate change is inextricably linked to air pollution, wherein one cannot be resolved without addressing the other^6^. Air pollution is the largest contributor to the burden of disease from the environment; seven million people die every year due to air pollution, with 90% of them in low- and middle-income countries^7^. Some air pollutants, such as black carbon^8^ and tropospheric ozone^9^, are also short-lived climate pollutants, responsible for a significant portion of air pollution-related deaths^10^. More than one million premature deaths are associated globally each year with high levels of ozone pollution^11^.

Heatwaves and ozone episodes share common underlying meteorological drivers and thus frequently coincide^10^. The oxidation of volatile organic compounds (VOCs) and carbon monoxide (CO) in the presence of nitrogen oxides (NOx) produces surface ozone, with increased reactivity at higher temperatures^12^ This is why surface temperatures and tropospheric ozone concentrations are strongly correlated^12,13^. Heatwaves and ozone extremes cluster together in often overlapping, multiday, spatially connected episodes^14,15^.

While no one is safe from climate-pollution-related risks, those whose health is being harmed first and worst are often people in low-income and disadvantaged countries and communities^15^. Prior efforts led by environmental justice researchers, who assess social inequalities in exposures to various hazards, have shown that socially vulnerable groups generally experience greater exposures to extreme heat^16–21^. Worsened by ground-level ozone, heatwaves affect the social and environmental determinants of health and disproportionally impact vulnerable populations around the world^15^. Although compound or concurrent heat-ozone extremes likely lead to death and illness, it remains challenging to assess their impact on different socioeconomic strata (affluent versus deprived population, for example).

Here, we have combined existing datasets of social indicators and climate-sensitive health risks with weather and air quality observations to assess health impacts of compound heat-ozone hazards in Santiago de Chile, a major mid-latitude Andean city of 7.7 million inhabitant. The city is entirely in the country's central valley and lies between 500 and 650 m above sea level (a.s.l), a region where heatwaves and extreme temperatures have surged in recent years^3^. The complicated surrounding topography (Fig. S1) as well as the regional subsidence thermal inversion layer^21^ and the relatively weak surface airstream increase the near-ground lower-tropospheric stability and constrain the dispersion of air pollutants^22^.

Due to poor air quality, too many people breath unhealthy air in Santiago on too many days of the year^23^. Most of the population lives in zones with unhealthy levels of ozone in summer and particle pollution in winter (Fig. S2). Both the 24-h mean concentration of particles with diameter of 2.5 µm or less (PM2.5) and the daily maximum 8-h mean ozone concentration often exceed levels recommended by the World Health Organization (WHO) (15 µg/m^3^ for PM2.5 and 100 µg/m^3^ for ozone)^24^ as well as levels stated in the more permissive Chilean regulations (20 µg/m^3^ for PM2.5 and 120 µg/m^3^ for ozone)^25,26^. Millions of Chileans are exposed to more than 40 unhealthy ozone days every year^9^.

Santiago is also a highly segregated city with distinct zones of affluence and deprivation^27^. This setting offers an opportunity for studying the effects of compound heat-ozone extremes on different socioeconomic strata. Disparities in exposure to climate-pollution-related risks are frequently worsened by the lack of access to health care affecting disadvantaged populations^27^ and by other social determinants of health like unhealthy diet and obesity^28^. In the case of Chile, the deprived population exhibits an overall less healthy diet than affluent dwellers^29^. Chile has the third-highest obesity rate among the Organization for Economic Cooperation and Development (OECD), with higher rates reported among women and socioeconomically marginalized communities^30^.

Attributable to spatial variations in the ground-level ozone burden (heavier for wealthy communities), we found that the response (i.e., mortality) to extreme heat (and the associated further ozone pollution) is stronger in affluent dwellers, regardless of comorbidities and inequities in the access to health care. Our findings underline the need for a site-specific hazard assessment and for a community-based risk management.

## Results

**Figure 1:**
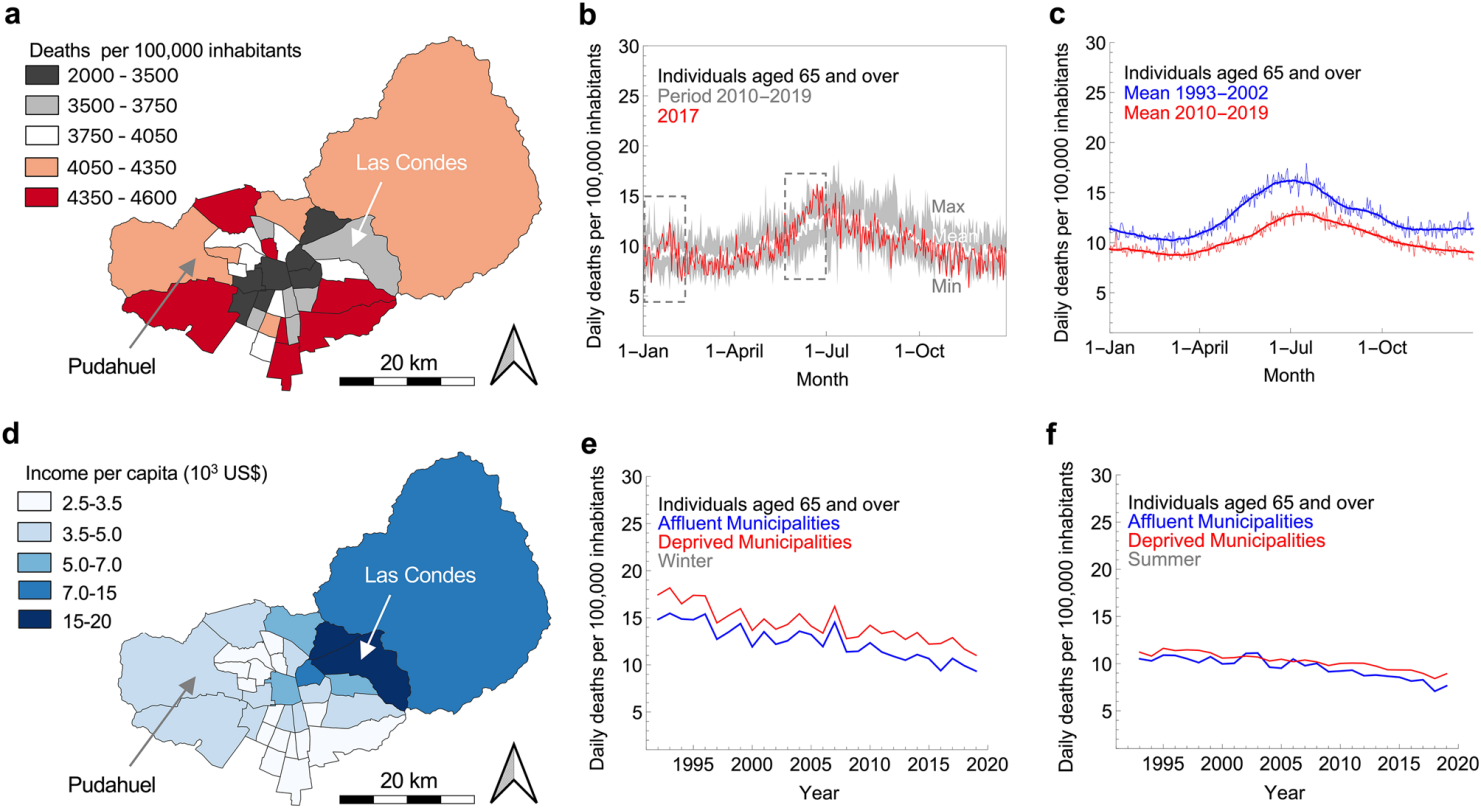
While socioeconomic inequalities generally drive disparities in the mortality rate, the gap between rich and poor considerably narrows during summer. (**a**) Annual mortality rate (number of annual deaths per 100,000 population) in individuals aged 65 and over across Santiago, averaged over the period 2010–2019. Mortality from all causes was used in this study. Among the more than 50 municipalities of Santiago, central and northeastern municipalities generally exhibit considerably fewer annual deaths than other municipalities on city outskirts. Despite the larger share of older population in affluent municipalities, deaths of adults (aged 65 + years) per 100,000 population are on an annual basis considerably larger in deprived municipalities. (**b**) Daily mortality rate in inhabitants (aged ≥ 65 years) of Santiago. The gray shading indicates the highest and lowest rates for each day of year (DOY) over the period 2010–2019 while the white line indicates the mean over the same period. The daily mortality rate for 2017 is also shown (red line). The dotted rectangular boxes highlight two periods of considerable excess deaths in 2017, that are likely related to the extremely warm January 2017 and to an outbreak of Influenza A (H3N2) Variant Virus^33^. (**c**) Daily mortality rate in inhabitants (aged ≥ 65 years) of Santiago, averaged over two periods: 1993–2002 (blue line) and 2010–2019 (red line). Bold lines correspond to the 30-day centered moving averages. (**d**) Annual income per capita (2017 US$) across Santiago. Inhabitants of central and northeastern municipalities are considerably wealthier than inhabitants of municipalities on city outskirts. (**e**) Progress of winter mortality rate in inhabitants (aged ≥ 65 years) of affluent (blue line) and deprived (red line) municipalities over the period 1992–2019. Although mortality rates in adults (aged ≥ 65 years) have been reduced by about 30% over the last three decades, the gap between wealthy and disadvantaged adults (aged ≥ 65 years) remains during winter. (**f**) Progress of summer mortality rate in inhabitants (aged ≥ 65 years) of affluent (blue line) and deprived (red line) municipalities over the period 1992–2019. The gap between wealthy and disadvantaged adults (aged ≥ 65 years) narrows considerably during summer. Annual household income data are from the National Socioeconomic Characterization Survey^47^. The mortality rate is based on data from the Department of Statistics and Health Information^48^ and the National Statistical Institute^49^. In the case of Figs. e–f, municipalities of Santiago were clustered into two groups according to the socioeconomic status of their inhabitants (Table S1). Inhabitants of affluent municipalities are about 15% of the total population. Plots were generated using Python’s Matplotlib library^50^.

Disparities between communities in the exposure to unhealthy air and in the quality of their health care delivery system became apparent when comparing the annual mortality rate (i.e., the number of annual deaths per 100,000 population). Santiago extends into more than 30 municipalities (often referred to as *communes*). In this highly segregated city, higher-income population (about 15% of the total population) typically resides in the TOP6 higher socioeconomic status municipalities, which are all located in northeastern Santiago. Northeastern municipalities, considerably wealthier than other municipalities on city outskirts, generally exhibit considerably fewer annual deaths of adults (65 years and older) (Fig. 1a). Annual mortality rate in inhabitants (aged ≥ 65 years) of the poorest municipalities can be twice as high as the mortality rate in inhabitants (aged ≥ 65 years) of the richest municipalities. Despite these municipality-level differences, clean air initiatives in Santiago nowadays heavily rely on the national government for implementation, often ignoring the role of local governments and municipalities (that have shown elsewhere to be a key partner in air quality management^31,32^.

The signal of excess deaths associated with extreme temperatures has emerged in Santiago. The dotted rectangular boxes in Fig. 1b highlight two periods of considerable high mortality rates in 2017. Hundreds of excess deaths (defined as the difference between observed and expected deaths) occurred in January 2017 and in June 2017. While excess deaths in June 2017 have been attributed to an outbreak of influenza A (H3N2) variant virus^33^, excess deaths in January 2017 are likely related to the extremely warm austral summer 2016–2017 (the warmest observed before the COVID pandemic). The footprints of the extreme temperatures in January 2017 and of the H3N2 variant outbreak in June 2017 are also apparent when all age strata are considered (Fig. S3). The signal of excess deaths associated with extreme temperatures emerged relatively late in Santiago compared to major cities in the Northern Hemisphere; thousands of deaths have been attributed to historic heatwaves in Chicago in 1995^34^ and in central Europe in 2003^35^.

Increases in the number of deaths associated with global warming may have so far been partially masked by the remarkable drop in mortality rates observed during the last decades in Santiago (Fig. 1c). From 1990 to 2019 mortality rates in inhabitants (aged ≥ 65 years) of Santiago (regardless of the socioeconomic status) have been reduced by about 30% (Fig. 1e,f). Over the same period, Chile has experienced unprecedented economic growth and poverty reduction^36^. Although Chile is since 2010 an OECD member, a group of 38 countries including the most developed in the world^37^, high levels of income and health care inequalities persist^38^.

Affluence and deprivation likely play a major role in disparities in the mortality rate between rich and poor inhabitants in Santiago. During winter, the deaths of adults (aged 65 + years) per 100 000 population are about 20% higher in deprived municipalities than in affluent municipalities. Economic growth and poverty reduction over the last 3 decades have done little to close this gap (Fig. 1e), favorable to wealthy communities despite their largest share of senior citizens. There is a clear shift towards older ages (i.e., population ageing) in the distribution of the population of affluent municipalities; the share of older people is about 50% larger in affluent municipalities than in deprived municipalities.

While health care and income inequalities generally drive disparities in the mortality rate^27^, this gap narrows considerably during summer in the case of individuals aged 65 + . As shown in Fig. 1f, deaths of inhabitants (aged 65 + years) per 100 000 population are roughly the same during summer in affluent and deprived municipalities, regardless of health care inequalities^38^. The gap between rich and poor does widen again when younger strata are considered. The mortality rate in population younger than 65 years is about 20% higher in deprived municipalities than in affluent municipalities, regardless of the season (Fig. S4).

The narrowing of gaps in mortality rates between wealthy and disadvantaged inhabitants (aged ≥ 65 years), from about 20% in winter to about 5% in summer, suggests the influence of factors beyond the socioeconomic strata. These factors probably include heat and pollution. Extreme temperatures have become more frequent in the region in recent decades^3^ and Santiago’s air quality remains poor^23^. Thus, spatial variations in the heat-pollution burden (unfavorable for wealthy communities) likely explain why mortality rates gaps (between wealthy and disadvantaged individuals aged 65 +) close in summer (Fig. 1f).

**Figure 2:**
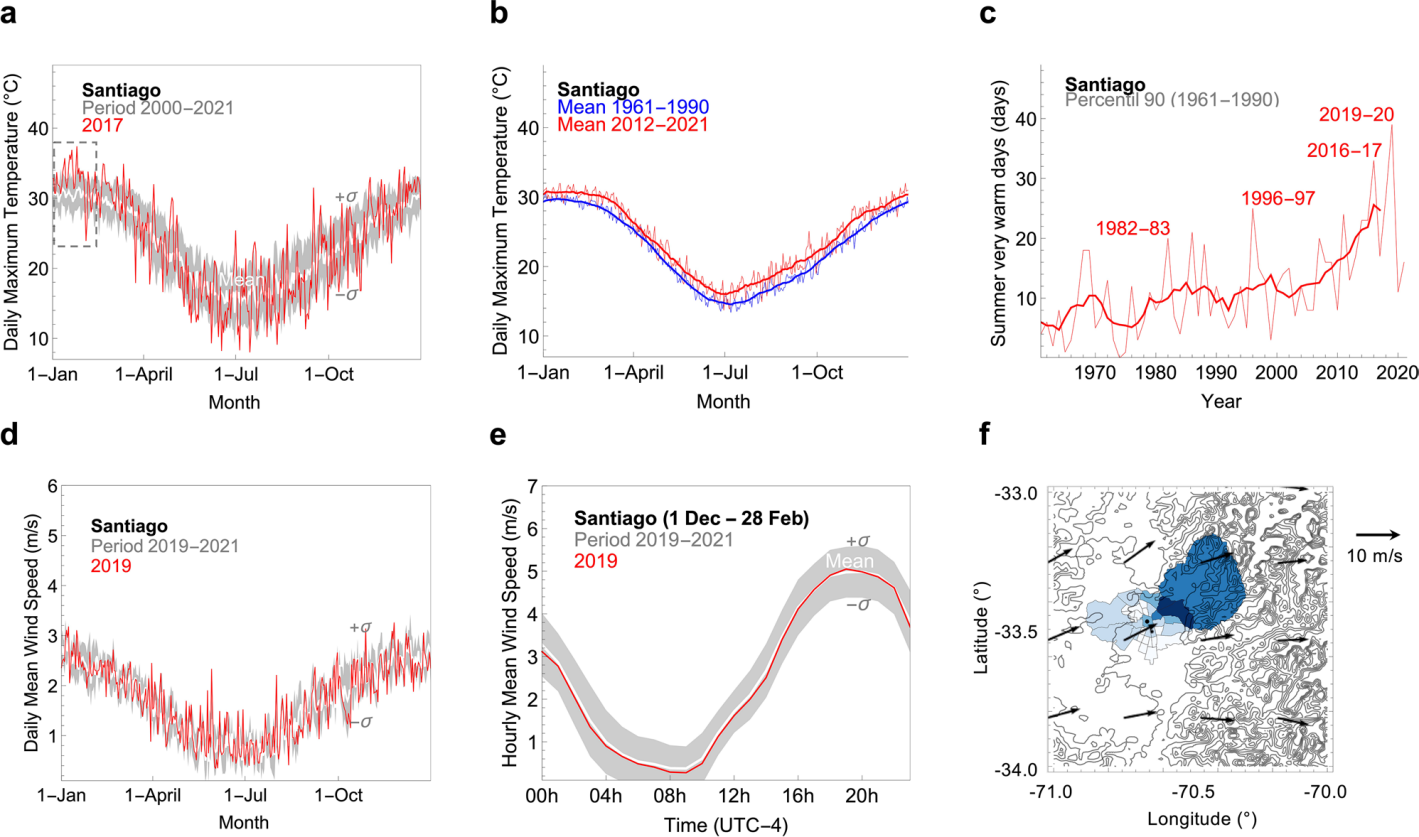
While rising temperatures affect all inhabitants, wind direction makes the ozone pollution burden heavier for wealthy northeastern communities. (**a**) Daily maximum temperature in Santiago. For each day of year (DOY), we formed datasets using daily maximum temperatures over the period 2000–2021. The mean (white line) and standard deviation (bounds of the gray shading) of these datasets are shown in the plot. The daily maximum temperature for 2017 is also shown (red line). The dotted rectangular box highlights a period of very warm days in January 2017. (**b**) Daily maximum temperature in Santiago averaged over two periods: 1961–1990 (blue line) and 2012–2021 (red line). Bold lines correspond to the 30-day centered moving averages. (**c**) Progress of summer ‘very warm” days in Santiago over the period 1961–2021. Bold line corresponds to the 7-year centered moving averages. We consider a summer day to be “very warm” if the corresponding maximum temperature falls above the 90th percentile of the daily base climatology (built up by using daily maximum temperatures measured over a 30-year base period 1961–1990; see “Methods”). (**d**) Daily average wind speed in Santiago. For each day of year (DOY), we formed datasets using values of the daily average wind speed over the period 2019–2021. The mean and standard deviation (σ) of these datasets are shown in the plot. The daily average wind speed for 2019 is also shown (red line). (**e**) Hourly average wind speed in Santiago. For each hour, we formed datasets using values of the hourly average wind speed over the period 2019–2021. The mean (white line) and standard deviation (bounds of the gray shading) of these datasets are shown in plots. (**f**) Vector array map showing 10-m summer wind speed and direction averaged from 13 to 20 h UTC over the period 2011–2020. Air temperature and wind speed are from the weather station that the Chilean Weather Service (DMC) operates since early twentieth century downtown Santiago; measurements are available at https://climatologia.meteochile.gob.cl/application/diario/visorDeDatosEma/330020. In the case of the vector array map, data from ERA5^46^ were used. Plots were generated using Python’s Matplotlib library^50^.

Heatwaves have surged in recent years in Santiago. During the extremely warm austral summer 2016–2017, the warmest observed before the COVID pandemic, five intense heatwaves pushed the daily maximum temperature well above usual conditions (Fig. 2a), likely contributing to hundreds of excess deaths in Santiago in early 2017 (Fig. 1b). While mean temperatures increased by about 1 °C over the last four decades (Fig. 2b), summer “very warm” days (Fig. 2c) and summer heatwaves (Fig. S5) more than doubled over the same period. Here, we consider a heatwave as a period of at least 3 consecutive “very warm” days^39^. A summer day is considered to be “very warm” if the corresponding maximum temperature falls above the 90th percentile of the daily base climatology (built up by using daily maximum temperatures measured over a 30-year base period 1961–1990; see “Methods” section).

The rise of “very warm” days alone cannot explain the closure of gaps in summer mortality rates between wealthy and disadvantaged inhabitants (aged ≥ 65 years). Since access to adequate cooling mechanisms is generally out of reach for vulnerable populations on city outskirts, rising temperatures are expected to widen the gaps between rich and poor^38^. In addition, there are considerable inequities in the city’s tree canopy cover (favorable for wealthy municipalities)^40^. Urban tree cover helps to mitigate the effects of urban heat islands through factors including evapotranspiration and shade^40^. Therefore, wealthy inhabitants of northeastern municipalities endure slightly lower extreme temperatures than disadvantaged inhabitants of western municipalities (Fig. S6).

While spatial variations in the temperature across the city may slightly favor affluent dwellers of northeastern municipalities, that is not always the case when it comes to air pollution. Dispersion and transport of pollutants by winds are strongly constrained by surrounding topography. In Santiago, wind speed is strongly season dependent (Fig. 2d) and considerably changes over the course of the day (Fig. 2e). In summer, strong winds that blow predominantly eastward (Fig. 2f) lower pollutant concentrations, but mostly over western municipalities. Blocked by the Andes (Fig. S1), pollutants tend to accumulate over affluent northeastern communities (Fig. S7). Hence, in summertime, wealthy inhabitants of northeastern municipalities endure considerably higher concentrations of ozone than disadvantaged inhabitants of western municipalities. The landscape influence on distribution of the pollution burden has been also observed in major cities in California^41^.

**Figure 3:**
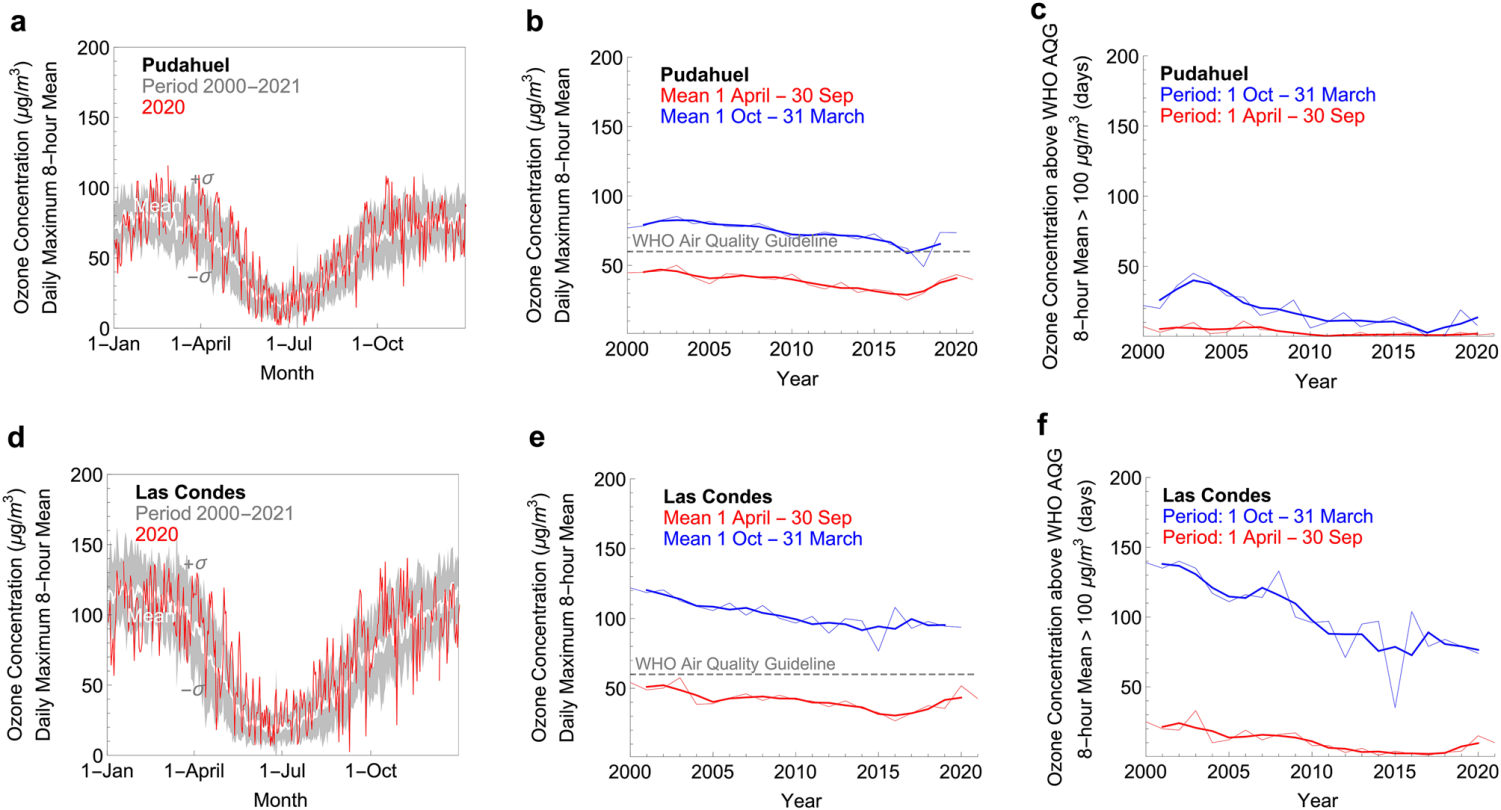
Ground-level ozone burden is considerably lower at Pudahuel (located on the deprived west side of the city) than at Las Condes (located in the affluent northeastern Santiago). (**a**) Daily maximum 8-h mean ozone concentration at Pudahuel station (33.44° S, 70.75° W, 460 m a.s.l., western Santiago). For each day of year (DOY), we formed datasets using daily maximum 8-h mean ozone concentrations over the period 2000–2021. The mean (white line) and standard deviation (bounds of the gray shading) of these datasets are shown in the plot. The daily maximum 8-h mean ozone concentration for 2017 is also shown (red line). (**b**) Progress of daily maximum 8-h mean ozone concentration at Pudahuel station (western Santiago) averaged over the period 1 April–30 Sep (red line) and over the period 1 Oct–31 March (blue line). Bold lines correspond to the 3-year centered moving averages. The dotted gray line indicates the 6-month peak season safe level (60 µg/m^3^) according to the air quality guidelines (AQG) by the World Health Organization (WHO)^24^. (**c**) Number of days at Pudahuel station (western Santiago) with a daily maximum 8-h mean ozone concentration higher than 100 µg/m^3^ (which is the daily maximum 8-h mean safe level according to the WHO^24^. Bold lines correspond to the 3-year centered moving averages. (**d**) Daily maximum 8-h mean ozone concentration at Las Condes station (33.38° S, 70.52° W, 795 m a.s.l., northeastern Santiago). For each day of year (DOY), we formed datasets using daily maximum 8-h mean ozone concentrations over the period 2000–2021. The mean (white line) and standard deviation (bounds of the gray shading) of these datasets are shown in the plot. The daily maximum 8-h mean ozone concentration for 2017 is also shown (red line). No measurements were conducted in February 2017 due to maintenance of the station. (**e**) Progress of daily maximum 8-h mean ozone concentration at Las Condes station (northeastern Santiago) averaged over the period 1 April–30 Sep (red line) and over the period 1 Oct–31 March (blue line). Bold lines correspond to the 3-year centered moving averages. The dotted gray line indicates the 6-month peak season safe level (60 µg/m^3^) according to the WHO^24^. (**f**) Number of days at Las Condes station (northeastern Santiago). with a daily maximum 8-h mean ozone concentration higher than 100 µg/m^3^ (which is the daily maximum 8-h mean safe level according to the WHO^24^. Bold lines correspond to the 3-year centered moving averages. Ozone measurements are from the air quality monitoring network operated by the Chilean Ministry of Environment (MMA) available at: https://sinca.mma.gob.cl/index.php/region/index/id/M. Plots were generated using Python’s Matplotlib library^50^.

According to ground-based measurements, ozone pollution burden is considerably heavier for wealthy inhabitants of northeastern municipalities in Santiago. Figure 3 compares ozone concentrations measured over the last two decades at two stations: Pudahuel (located on the deprived west side of the city) and Las Condes (located in the affluent northeastern Santiago); Fig. 1a indicates locations of both stations. During the ozone low season (1 April–30 Sep) ground-level ozone concentrations at Las Condes and Pudahuel are comparable. However, during the ozone peak season (1 Oct–31 March), the daily maximum 8-h mean ozone concentration is about 35% higher at Las Condes station than at Pudahuel station (Fig. S8). Differences between Pudahuel and Las Condes are also noticeable when comparing the number of days at Pudahuel station with a daily maximum 8-h mean ozone concentration higher than 100 µg/m^3^, which is the daily maximum 8-h mean safe level recommended by the WHO^24^. During the peak season, daily maximum 8-h mean ozone concentrations exceed the 100 µg/m^3^ safe level about 4 times more frequently at Las Condes than at Pudahuel (compare blue lines in Fig. 3c,f). The spatial variations in the ozone burden across Santiago are extensively discussed by Seguel et al.^9^.

Driven by air-quality regulations, progress bringing down ozone pollution and cleaning the air has likely played a role in the remarkable drop in mortality observed during the last decades (Fig. 1f). Yet, Santiago has a long way ahead regarding ozone pollution. Although ozone concentrations in affluent and deprived municipalities have declined by about 20% over the last two decades^9^, the daily maximum 8-h mean ozone concentration averaged over the period 1 Oct–31 March is still higher than 60 µg/m^3^, which is the 6-month peak season safe level according to the WHO^24^.

Especially for richer northeastern communities, ozone pollution considerably worsens the effects of increasingly warm summers in Santiago. Heatwaves and multiday ozone episodes also share common underlying meteorological drivers^10^. Ground-level ozone is more readily produced on warm and sunny days that favor photochemical reactions that form ozone. Warmer temperatures increase emissions of biogenic VOCs and make nitrous oxide lifetime longer, which augment the production of surface ozone. Therefore, as also observed elsewhere^12,13^, summer temperatures and tropospheric ozone concentrations in Santiago are correlated (Figs. S9–10).

**Figure 4:**
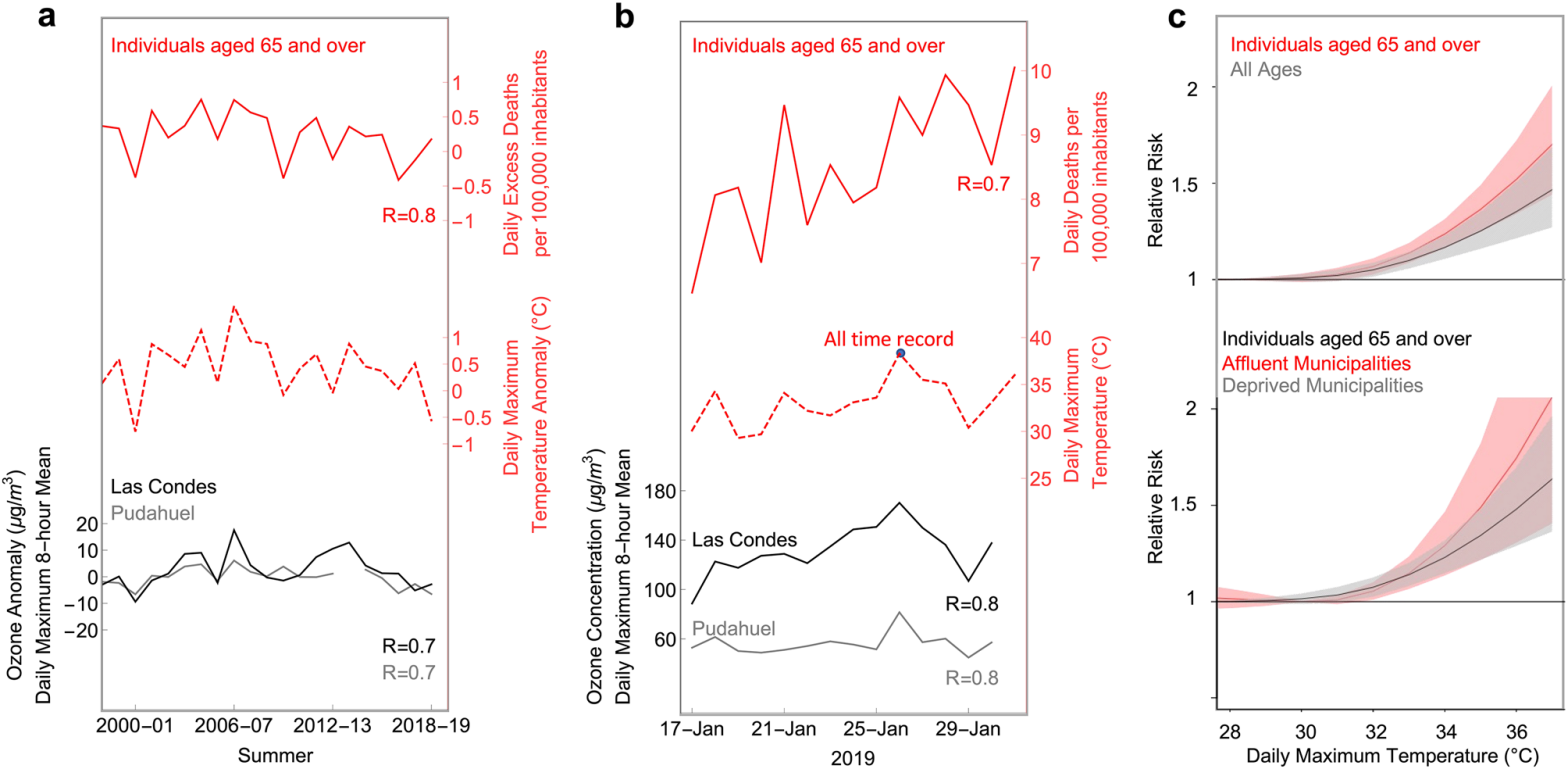
The rise in the mortality risk associated with heat-ozone compound extremes is larger in affluent communities than in deprived communities in Santiago. (**a**) Progress of daily excess deaths in adults (aged ≥ 65 years, all socioeconomic strata; upper red line), daily maximum temperature anomalies (dotted red line), and the anomalies of daily maximum 8-h mean ozone concentration at Las Condes station (black line) and at Pudahuel station (gray line). For detrend purposes, daily anomalies and daily excess deaths were computed using the summer average of each year as a reference. Then, daily anomalies and daily excess deaths were averaged over the 30-day warmest period (20 Dec–18 Jan) of the year. The correlation coefficients (R) between the temperature anomaly and the excess deaths (as well as the ozone concentration anomalies) are shown in the plot. (**b**) Progress of the daily mortality rate in adults (aged ≥ 65 years, all socioeconomic strata; upper red line), the daily maximum temperature (dotted red line), and the daily maximum 8-h mean ozone concentration at Las Condes station (black line) and at Pudahuel station (gray line), over the period 17 Jan 2019 and 30 Jan 2019. The correlation coefficients (R) between the temperature and the mortality rate (as well as the ozone concentration) are shown in the plot. Santiago hit its all-time heat record (38.3 °C) on 26 Jan 2019. (**c**) Heat-mortality associations for two age strata (upper plot) and for two socioeconomic strata (lower plot). Mortality from all causes was used in this study. Exposure–response associations are estimated as best linear unbiased predictions (see “Methods”) and reported as Relative Risks. Bold lines represent the risks (shading indicates 95% confidence intervals) of exposure to a daily maximum temperature, relative to the risk corresponding to the temperature of minimum mortality (which in Santiago is about 27 °C). In Santiago, the 99th percentile of the daily maximum temperature (summer) is 35 °C. Plots were generated using Python’s Matplotlib library^50^.

Existing datasets suggest that positive compound heat-ozone anomalies increase the mortality rate in adults (aged 65 + years). Especially over the 30-day warmest period (20 Dec–18 Jan) of the year in Santiago, warmer summers are correlated with excess deaths in adults (aged 65 + years) (Fig. 4a). The highest temperature ever recorded in Santiago was 38.3 °C, on 26 Jan 2019, as the region was hit by a heatwave that began a few days before (Fig. 4b). These extreme temperatures triggered a multiday ozone episode, which predictably pushed the daily maximum 8-h mean ozone concentration at Las Condes station well above the 100 µg/m^3^ safe level (Fig. 4b). These compound or concurrent heat-ozone extremes likely contributed to the rise in the mortality rate of adults (aged 65 + years) observed in Santiago in late January 2019 (Fig. 4b).

However, what makes the case of Santiago particularly interesting is that compound heat-ozone extremes roughly equally affect city dwellers (aged ≥ 65 years) regardless of their socioeconomic status. In other words, the mortality burdens associated with the additional heat exposure (and the associated further ozone pollution) are approximately equally divided between rich and poor inhabitants of Santiago (Fig. 1f). Concurrent warm temperatures and high ozone concentrations likely explain the narrowing of gaps in mortality rates between wealthy and disadvantaged adults (aged ≥ 65 years), from about 20% in winter (Fig. 1e) to about 5% in summer (Fig. 1f).

Considering comorbidities and lack of access to health care affecting disadvantaged population^27^, the narrow gap in summer mortality rates between rich and poor (Fig. 1f) can only be explained by a stronger response of wealthy inhabitants to extreme heat (and the associated enhanced ozone pollution). This is confirmed by exposure–response associations obtained by applying regression techniques^42,43^ to available temperature and mortality data. The exposure–response associations characterize the complex relationship between temperature and mortality (all causes), account for the nonlinear and delayed dependencies (see “Methods”) and are reported as Relative Risks. Bold lines in Fig. 4c represent the risks of exposure to a daily maximum temperature, relative to the risk corresponding to the temperature of minimum mortality.

While the response (i.e., the mortality) to extreme heat (and the associated further ozone pollution) is considerable regardless of age and income, it is stronger in the case of adults (aged ≥ 65 years) of affluent municipalities (Fig. 4c). Relative to the risk corresponding to the temperature of minimum mortality, the mortality risk in adults (aged ≥ 65 years, all socioeconomic strata) increases by about 30% when the daily maximum temperature reaches 35 °C (upper panel). The latter temperature (corresponding to the 99th percentile of the daily maximum temperature) is associated with a 50% larger mortality risk in wealthy inhabitants (aged ≥ 65 years) of northeastern municipalities (lower panel).

## Discussion

Rising temperatures are worsening ozone pollution in Santiago, which is already undergoing the so-called “climate penalty”. Dwellers of this highly segregated city are enduring concurrent heatwaves and ozone episodes, which are likely leading to death and illness. Here, we have combined existing datasets of social indicators and climate-sensitive health risks with weather and air quality observations to study the response of different socioeconomic strata to compound heat-ozone extremes.

Affluence and deprivation drive inequalities in the mortality rate between the more than 30 municipalities of Santiago. Annual deaths of adults (aged 65 + years) per 100 000 population are two times larger in deprived municipalities than in affluent municipalities. While disparities in social determinants of health generally contribute to inequalities in the mortality rate, this gap narrows considerably during summer in the case of adults (aged 65 + years).

The unexpected narrow gap in summer mortality rates between rich and poor in Santiago (Fig. 1f) likely results from spatial variations in the temperature and ground-level ozone concentrations. While spatial variations in the temperature across the city may slightly favor affluent dwellers of northeastern municipalities, that is not the case when it comes to air pollution. Ground-level ozone pollution burden is considerably heavier for wealthy inhabitants of northeastern municipalities in Santiago.

Attributable to spatial variations in the temperature and surface ozone concentrations, we found that compound heat-ozone extremes roughly equally affect city dwellers (aged 65 + years) regardless of the socioeconomic status. In other words, the mortality burdens associated with the additional heat exposure (and the associated further ozone pollution) are approximately equally divided between rich and poor inhabitants of Santiago (Fig. 1f).

We also found that the response (i.e., the mortality) to extreme heat (and the associated further ozone pollution) is stronger in wealthy communities, regardless of comorbidities and lack of access to health care affecting disadvantaged population. The stronger response in wealthy communities is what makes the gap in mortality rates narrow from about 20% in winter (Fig. 1e) to about 5% in summer (Fig. 1f). Although the associated death tolls are higher in deprived municipalities, the relative risks to city dwellers of rich northeastern municipalities are greater. Due to higher ozone concentrations, residents in affluent northeastern municipalities may currently have a higher death risk per unit of temperature increase than residents in deprived municipalities.

We expect the narrow gap in summer mortality rates between rich and poor to widen in the future as, driven by air-quality regulations, Santiago brings down ozone pollution. Surface ozone concentrations across the city exhibit a trend of about − 10% per decade, which may partially offset the effect on the mortality of rising temperatures. As ozone concentrations fall, heatwaves become more frequent and more intense^3^. Air quality improvements will make the role of the temperature (and socioeconomic inequities) progressively more important when it comes to compound heat-ozone extremes. When exposed to similar heat-ozone conditions, lower-income adults are presumably more likely to die than wealthy city dwellers.

Our findings underline the urgent need for more ambitious adaptation strategies to minimize the public health impacts of compound heat-ozone extremes. For example, the development of a Heat/Health warning system (HHWS) appears to be urgently needed. Following the recommendations of the WHO and the World Meteorological Organization (WMO) warnings should be based on health effects, not just on air temperature. In Chile, authorities issue public alerts in case of heatwaves. However, these alerts are only based on temperature percentile and do not include additional parameters such as, for example, heat-mortality associations (Fig. 4c).

Climate penalties pose serious challenges to the global south due to its vulnerability (determined by population density, percentage of the low-income population and their spatial distribution) and its limited adaptive capacity (determined by limited access to information/resources and a weak institutional framework/governance). These challenges underline the importance of this effort. We expect that, by leading to an improved understanding of the impacts of compound heat-ozone extremes on human health, we will ultimately contribute to building more resilient and sustainable communities in the global south.

## Methods

### Observations and satellite retrievals

Air-quality measurements, including hourly ozone concentrations, are from the air quality monitoring network operated since the late 1990s by the Chilean Ministry of Environment (MMA). Ozone measurements are carried out by using an ozone analyzer Thermo Scientific Inc. (Model 49i).

According to the Air Quality Guidelines (AQG) by the World Health Organization (WHO)^24^:

- Daily maximum 8-h mean ozone concentrations exceeding 100 µg/m^3^, and
- Averages of daily maximum 8-h mean ozone concentrations during the six months with the highest 6-month running average of ozone concentration (roughly 1 Oct–1 March in Santiago) exceeding 60 µg/m^3^,

are of concern. As a reference, the level of concern considered in the more permissive Chilean regulation^26^ is 120 µg/m^3^ (in the case of the daily maximum 8-h mean ozone concentration).

Air temperature and wind speed are from the weather station operated downtown Santiago by the Chilean Weather Service (DMC) since the early twentieth century. Here, we considered a heatwave as a period of at least 3 consecutive “very warm” days. A summer day was considered to be “very warm” when its corresponding maximum temperature falls above the 90th percentile of the daily base climatology. The base climatology is based on the daily maximum temperatures measured over the base period 1961–1990. For each day of the year, we used a 15-day rolling window of the daily maximum air temperature over the base period, in order to form datasets of 450 values (15 days × 30 years). For each day of the year, these datasets of 450 values allowed us to compute the mean (that defined the daily base climatology) and the daily 90th percentile threshold^39^.

Satellite estimates of the land surface temperature and the tropospheric Nitrogen Dioxide (NO_2_) are based on Landsat-8 imagery^44^ and Sentinel-5P retrievals^45^. In the case of the vector array map (Fig. 2f), we used data from ERA5^46^.

### Socioeconomic status

Santiago is a highly segregated city with distinct zones of affluence and deprivation^27^. This setting offers an opportunity for studying the effects of concurrent heatwaves and ozone episodes on distinct zones of affluence and deprivation. Here, we clustered municipalities of Santiago into two groups according to the socioeconomic status of their inhabitants (Table S1). We considered affluent municipalities those in the TOP6 of the list of municipalities sorted by their socioeconomic status index, as defined by Mena et al.^27^. The socioeconomic status index computed from the social priority index (or “indice de prioridad social” in Spanish) reported annually by the Chilean Ministry of Social Development and Family. The social priority index is based on three factors: (i) income, (ii) access to education, and (iii) access to health care. Annual household income data in Fig. 1d are from the National Socioeconomic Characterization Survey^47^. In this highly segregated city, wealthy population typically resides in the TOP6 higher socioeconomic status municipalities, which are all located in northeastern Santiago.

Although Santiago is a highly segregated city, note that using municipalities for discerning socioeconomic strata has some limitations compared with studies that uses, for example, census tracts as analytical units (e.g., Renteria et al.^16^). These limitations suggest that future efforts aimed at the studying disparities in the exposure to environmental hazards in Santiago may need to advance to neighborhood-level information. In addition, although we clustered municipalities according to a social priority index (based on three factors: (i) income, (ii) access to education, and (iii) access to health care), future endeavors may also consider additional social indicators such as racial/ethnic minorities^17^, assess per households^18^, or number of inhabitants per household^19^.

### Mortality

We retrieved mortality data in Chile from the Department of Statistics and Health Information (DEIS by its Spanish acronym) for 1990–2019^48^. Population data was taken from the annual projection database by the National Statistical Institute (INE by its Spanish acronym)^49^. Mortality rate (number of annual deaths per 100,000 population) was computed for different age and socioeconomic strata.

### Heat-mortality associations

Exposure–response (heat-mortality) associations obtained by applying regression techniques^42,43^ to available temperature and mortality data. The exposure–response associations characterize the complex relationship between temperature and mortality (all causes), account for the nonlinear and delayed dependencies and are reported as Relative Risks. To account for non-linear, delayed effects of heat stress on mortality, we deployed a distributed lag non-linear model (DLNM) using the dlnm package (version 2.4.6) from R software^42^. Following the methodology used by Gasparrini et al.^43^, we used a quasi-Poisson distribution regression to compute the temperature–mortality relationship using the Relative Risk (RR). DLNM uses two functions: firstly, a natural cubic B-spline with three internal knots placed at the 10th, 75th, and 90th percentiles, a flag for day of the week, and 8 degrees of freedom per year to consider seasonal effects and long-term trends; secondly a cubic natural spline with three internal knots equally-spaced in the log-scale and a lag period of 21 days.

## Supplementary Information

Supplementary Information.

## Data Availability

The datasets and codes used and/or analyzed during the current study available from the corresponding author on request.
