# Supplementary material for: Compound climate-pollution extremes in Santiago de Chile
Source: Sci Rep. 2023 Apr 25;13:6726. doi: 10.1038/s41598-023-33890-w (PMC10130055; doi:10.1038/s41598-023-33890-w)
Supplement: Supplementary file 1 — Supplementary Information. [file 41598_2023_33890_MOESM1_ESM.pdf]

Supplementary Information

Compound Climate-Pollution Extremes in Santiago de Chile

Sarah Feron[1,2], Raúl R. Cordero[1,*], Alessandro Damiani[3], Pedro Oyola[4], Tabish Ansari[5], Juan C. Pedemonte[6], Chenghao Wang[7,8], Zutao Ouyang[9], Valentina Gallo[2]

1 Universidad de Santiago de Chile. Av. Bernardo O'Higgins 3363, Santiago, Chile.
2 University of Groningen, Wirdumerdijk 34, 8911 CE, Leeuwarden, The Netherlands.
3 Center for Environmental Remote Sensing, Chiba University, 1-33 Yayoicho, Inage Ward, Chiba, 263-8522, Japan.
4. Centro Mario Molina, Antonio Bellet 292, Santiago, Chile
5. Research Institute for Sustainability – Helmholtz Centre Potsdam (RIFS), Berliner Str. 130, 14467 Potsdam, Germany
6. School of Medicine, Pontificia Universidad Católica de Chile, Santiago, Chile
7. School of Meteorology & Department of Geography and Environmental Sustainability, University of Oklahoma, 120 David L. Boren Blvd. Suite 5220, Norman, OK 73072, USA
8. Department of Geography and Environmental Sustainability, University of Oklahoma, Norman, OK 73019, USA
9. Department of Earth System Science, Stanford University, Stanford, CA, 94305-2210, USA.

* Corresponding Author
Raúl R. Cordero
raul.cordero@usach.cl

The complicated surrounding topography constrains the dispersion of air pollutants

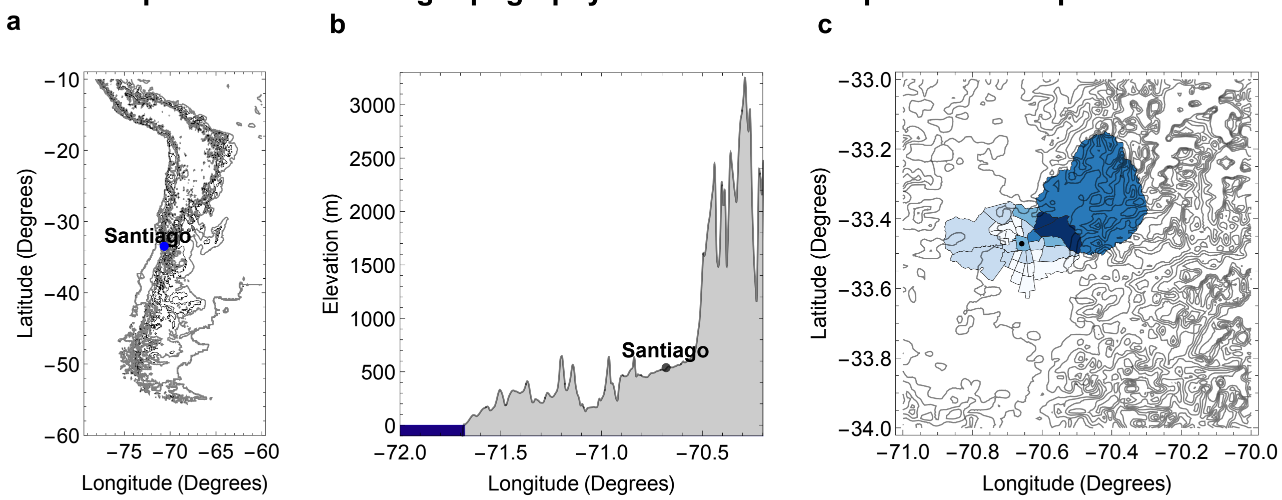

Fig. S1

a) Santiago is a major mid-latitude Andean city surrounded by a complicated topography.
b) Elevation along latitude 33°27’ S; the city lies between 500–650 m above mean sea level.
c) Santiago footprint in the Chilean central valley.

Based on the annual concentration of particles with diameter of 2.5 micrometres or less (PM2.5), Santiago ranks as one of the most polluted capital cities in the world.

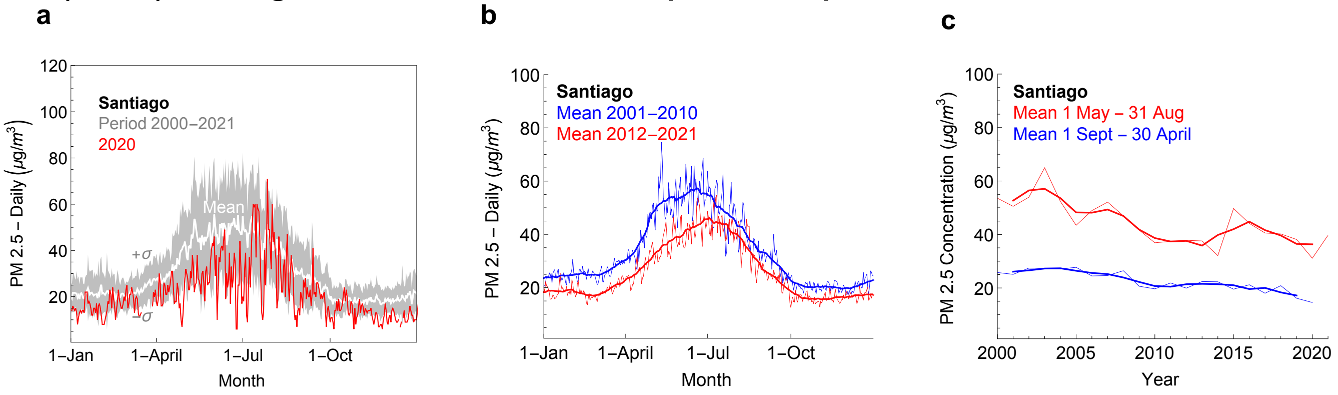

Fig. S2

a) Daily mean PM2.5 concentration at O'Higgins station (33.46ºS, 70.66ºW, 540 m a.s.l., downtown Santiago). For each day of year (DOY), we formed datasets using daily mean ozone concentrations over the period 2000–2021. The mean (white line) and standard deviation (bounds of the gray shading) of these datasets are shown in the plot. The daily mean PM2.5 concentration for 2020 is also shown (red line); the effect of the lockdowns related to the COVID pandemic are apparent.

b) Daily mean PM2.5 concentration at O'Higgins station (downtown Santiago) averaged over two periods: 2001–2010 (blue line) and 2012–2021 (red line). Bold lines correspond to the 30-day centered moving averages.

c) Progress of PM2.5 concentration at O'Higgins station (downtown Santiago) averaged each year over two periods: 1 May – 31 Aug (red line) and 1 Sep – 30 April (blue line). Bold lines correspond to the 3-year centered moving averages.

PM2.5 concentration measurements are from the air quality monitoring network operated by the Chilean Ministry of Environment (MMA) available at: https://sinca.mma.gob.cl/index.php/region/index/id/M.

Hundreds of excess deaths in January 2017 are likely related to the extremely warm austral summer 2016-2017.

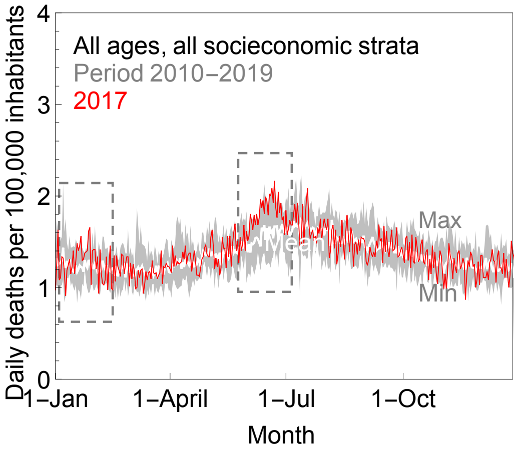

Fig. S3

Daily mortality rate in inhabitants (all ages, all socioeconomic strata) of Santiago. The gray shading indicates the highest and lowest rates for each day of year (DOY) over the period 2010–2019 while the white line indicates the mean over the same period. The daily mortality rate for 2017 is also shown (red line). The dotted rectangular boxes highlight two periods of considerable excess deaths in 2017.

Considering inhabitants younger than 65 years, the mortality rate gap between rich and poor exhibited few changes over the last three decades.

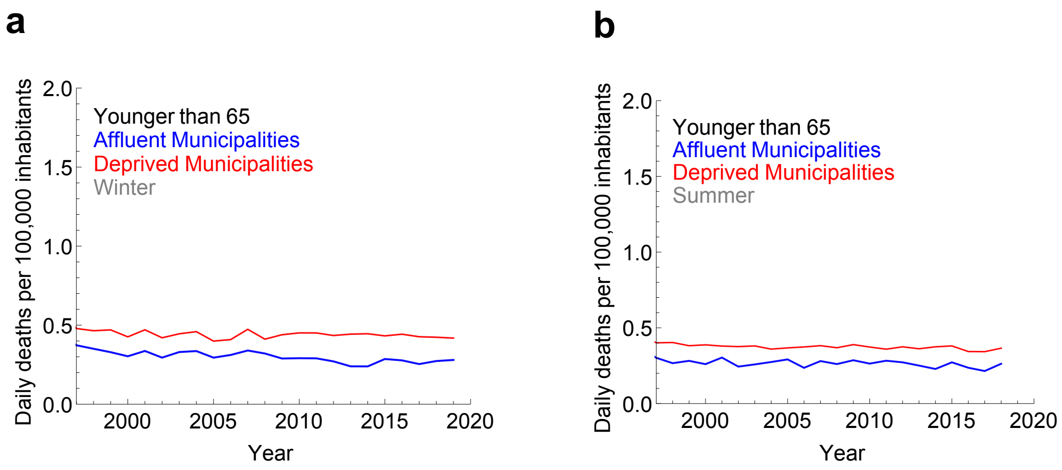

Fig. S4

a) Progress of winter mortality rate in inhabitants (younger than 65 years) of affluent (blue line) and deprived (red line) municipalities.

b) Progress of summer mortality rate in inhabitants (younger than 65 years) of affluent (blue line) and deprived (red line) municipalities.

Municipalities of Santiago were clustered into two groups according to the socioeconomic status of their inhabitants (Table S1). Inhabitants of affluent municipalities are about 15% of the total population.

Heatwaves have surged in the last two decades in Santiago

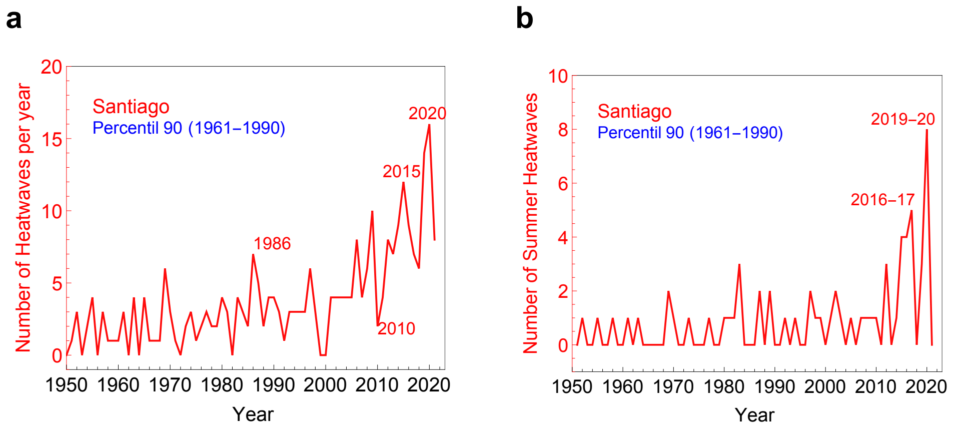

Fig. S5

Progress of heatwaves (defined as a period of at least 3 consecutive “very warm” days). A day is considered to be “very warm” if the corresponding maximum temperature falls above the 90th percentile of the daily base climatology (built up by using daily maximum temperatures measured over a 30-year base period 1961–1990; see “Methods”).

a) Number of heatwaves per year.
b) Number of heatwaves per summer.

Air temperature are from the weather station operated by the Chilean Weather Service (DMC), available at https://climatologia.meteochile.gob.cl/application/diario/visorDeDatosEma/330020.

Spatial variations in the land surface temperature likely arise from inequities in the city's tree canopy (favorable for richer municipalities).

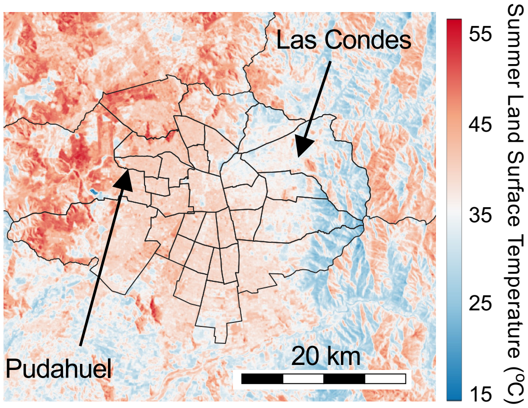

Fig. S6

Summer land surface temperature averaged over the period 2013-2021 derived from Landsat-8 imagery.

Blocked by the Andes, pollutants tend to pile up over the affluent northeastern municipalities in summer.

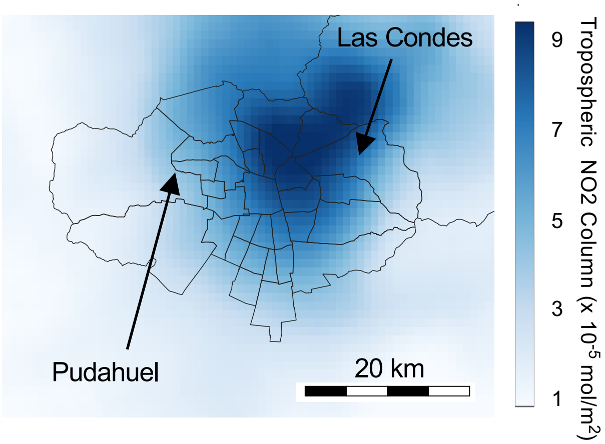

Fig. S7

Summer tropospheric Nitrogen Dioxide (NO2) averaged over the period 2018-2022.

Data were retrieved from Sentinel-5P data, available at https://sentinels.copernicus.eu/web/sentinel/data-products/-/asset_publisher/fp37fc19FN8F/content/sentinel-5-precursor-level-2-nitrogen-dioxide

Despite the spatial differences, ground-level ozone concentrations measured at different municipalities in Santiago are correlated.

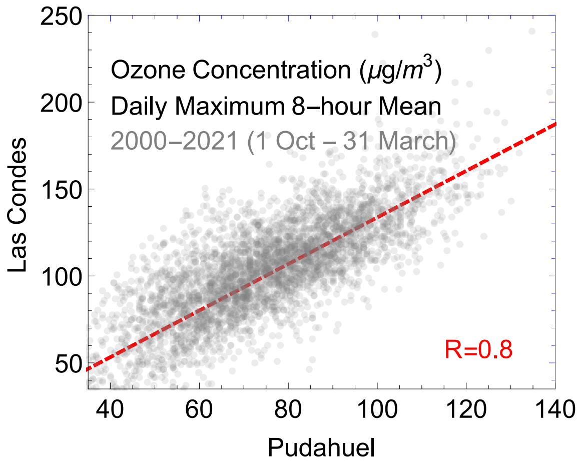

Fig. S8

Comparisons between the daily maximum 8-hour mean ozone concentrations measured at Las Condes station and at Pudahuel station, over the period 2000-2021. The correlation coefficient (R) is shown in the plot.

Ozone measurements are from the air quality monitoring network operated by the Chilean Ministry of Environment (MMA), available at: https://sinca.mma.gob.cl/index.php/region/index/id/M

Ground-level ozone concentration changes over the course of the day influenced, among other factors, by the air temperature.

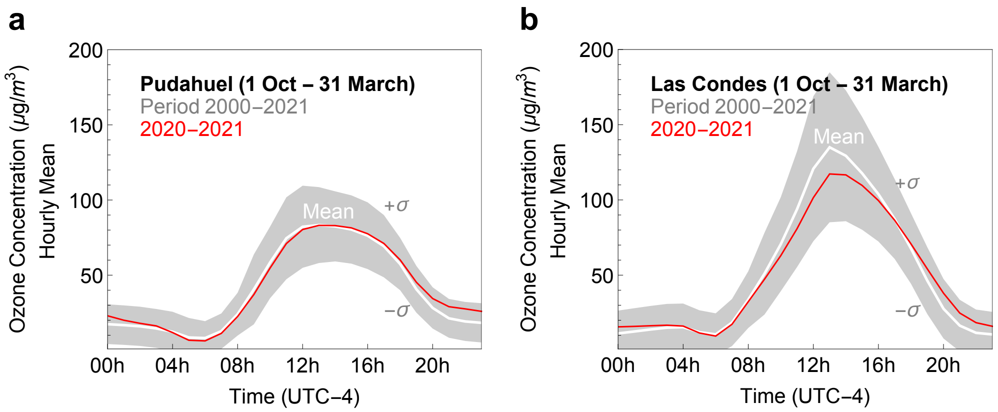

Fig. S9

a) Hourly mean ozone concentration at Pudahuel station (western Santiago). For each hour, we formed datasets using values of the hourly mean ozone concentration over the period 2000–2021. The mean (white line) and standard deviation (bounds of the gray shading) of these datasets are shown in the plots.

b) Hourly mean ozone concentration at Las Condes station (northeastern Santiago). For each hour, we formed datasets using values of the Hourly mean ozone concentration over the period 2000–2021. The mean (white line) and standard deviation (bounds of the gray shading) of these datasets are shown in plots.

Ozone measurements are from the air quality monitoring network operated by the Chilean Ministry of Environment (MMA), available at: https://sinca.mma.gob.cl/index.php/region/index/id/M

Surface temperatures and tropospheric ozone concentrations are strongly correlated.

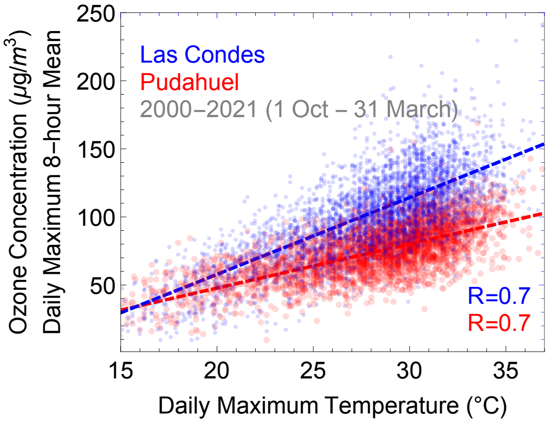

Fig. S10

Comparisons between daily maximum temperature measured downtown Santiago and the daily maximum 8-hour mean ozone concentration measured at Las Condes station (blue) and at Pudahuel station (red), over the period 2000-2021. The correlation coefficients (R) are shown in the plot.

Ozone measurements are from the air quality monitoring network operated by the Chilean Ministry of Environment (MMA), available at: https://sinca.mma.gob.cl/index.php/region/index/id/M Air temperature are from the weather station operated by the Chilean Weather Service (DMC), available at https://climatologia.meteochile.gob.cl/application/diario/visorDeDatosEma/330020

Table S1. Municipalities of Greater Santiago clustered into two groups according to the socioeconomic status of their inhabitants. We considered affluent municipalities those in the TOP6 of the list of municipalities sorted by their socioeconomic status index (see "Methods"). Inhabitants of affluent municipalities are about 15% of the total population. There is a clear shift towards older ages (i.e., population ageing) in the distribution of the population of affluent municipalities; the share of older people is about 50% larger in affluent municipalities than in deprived municipalities. Yet, annual mortality rate in inhabitants (aged ≥65 years) of the poorest municipalities can be twice as high as the mortality rate in inhabitants (aged ≥65 years) of the richest municipalities.

Affluent	Deprived	Deprived
Vitacura	Santiago	San Joaquín
Las Condes	Cerrillos	San Miguel
Providencia	Cerro Navia	San Ramón
Lo Barnechea	Conchalí	Puente Alto
La Reina	El Bosque	Pirque
Ñuñoa	Estación Central	San José de Maipo
	Huechuraba	Colina
	Independencia	Lampa
	La Cisterna	Tiltil
	La Florida	San Bernardo
	La Granja	Buin
	La Pintana	Calera de Tango
	Lo Espejo	Paine
	Lo Prado	Melipilla
	Macul	Alhué
	Maipú	Curacaví
	Pedro Aguirre Cerda	María Pinto
	Peñalolén	San Pedro
	Pudahuel	Talagante
	Quilicura	El Monte
	Quinta Normal	Isla de Maipo
	Recoleta	Padre Hurtado
	Renca	Peñaflor
